# Asymptomatic Bacteriuria and Antibacterial Susceptibility Patterns in an Obstetric Population

**DOI:** 10.5402/2011/721872

**Published:** 2011-01-24

**Authors:** Şevki Çelen, Ayla Sargin Oruç, Rana Karayalçin, Sibel Saygan, Serpil Ünlü, Belgin Polat, Nuri Danişman

**Affiliations:** ^1^Obstetrics and Gynecology, Zekai Tahir Burak Women's Health Education and Research Hospital, Ankara 06230, Turkey; ^2^Microbiology and Clinical Microbiology, Zekai Tahir Burak Women's Health Education and Research Hospital, Ankara 06230, Turkey; ^3^Infectious Disease and Clinical Microbiology, Zekai Tahir Burak Women's Health Education and Research Hospital, Ankara 06230, Turkey

## Abstract

*Introduction*. Asymptomatic bacteriuria (ASB), occurring in 2–11% of pregnancies, is a major predisposition to the development of pyelonephritis, which is associated with obstetrical complications, such as preterm labor and low birth weight infants. The aim of this study was to determine the prevalence of ASB, the antibacterial susceptibilities of the isolated microorganisms and the associated risk factors in an outpatient clinical setting in Zekai Tahir Burak Women's Health Education and Research Hospital in Ankara, Turkey. *Material and Methods*. Between December 2009 and May 2010, pregnant women admitted to the antenatal outpatient clinic were included in this study. The results of a complete urine analysis, midstream urine culture and antibacterial susceptibility were evaluated. *Results*. Of the 2011 pregnant women included, 171 had ASB (8.5%). *E. coli* was the most frequently isolated microorganism (76.6%), followed by Klebsiella pneumonia (14.6%). Both microorganisms were highly sensitive to fosfomycin, sensivity being 99.2% for *E. coli* and 88% for Klebsiella pneumonia. *Conclusions*. In this certain geographical region, we found *E. coli* as the most common causative agent of ASB in the obstetric population and it is very sensitive to fosfomycin. We recommend fosfomycin for ASB in pregnant women due to its high sensitivity, ease of administration and safety for use in pregnancy.

## 1. Introduction

Urinary tract infections (UTI) are the most common bacterial infections of pregnancy [1]. Asymptomatic bacteriuria (ASB), occurring in 2–11% of pregnancies, is a major predisposition to the development of pyelonephritis, which is associated with obstetrical complications, such as preterm labor and low birth weight infants. Bacteriuria is defined as the presence of >10^5^ colonies of a single pathogen per milliliter of urine. It may be either an asymptomatic bacteriuria (ASB of pregnancy) or symptomatic acute cystitis and acute pyelonephritis [2].

When left untreated, ASB may lead to symptomatic uriner tract infections (UTI) [3]. Untreated, ASB is found to be associated with subsequent acute pyelonephritis in 20–50% of cases [4].

The female urethra is relatively short and is anatomically proximal to the vagina, which is colonized with organisms from the gastrointestinal tract. Normal physiological changes in pregnancy place women at risk for pyelonephritis. There is also relative obstruction of the ureters because the enlarging uterus physically blocks them, and the hormonal milieu of pregnancy leads to relaxation of the smooth muscle of the ureters and the bladder. Furthermore, the glycosuria and aminoaciduria of pregnancy provide an excellent medium for bacterial proliferation [5–7].

The relatively high prevalence of ASB during pregnancy, the significant consequences for women and pregnancy, and the ability to avoid undesired outcomes with treatment justify screening and treatment of ASB in pregnancy [8].

The frequency of isolated pathogens and antimicrobial resistance patterns can vary in different geographical regions; therefore, the most common causative agents should be investigated and communities should be aware of their local antimicrobial resistances. The objective of this prospective study was to identify the prevalence of ASB, the most common causative agents and the antibacterial susceptibilities of the isolated microorganisms in a Research Hospital in Ankara, Turkey.

## 2. Materials and Method

Between December 2009 and May 2010, 2011 of 2132 pregnant women applying for their first antenatal visit to the Zekai Tahir Burak Women's Health Education and Research Hospital antenatal outpatient clinic were recruited into the study. The remaining 121 women refused to take part in this study, and informed consent for the participating patients was taken. Patients with fever, urinary symptoms (such as dysuria, hesitancy, urgency, frequent voiding, incontinence and incomplete voiding), current antibiotic therapy, and a history of urolithiasis and diabetes were excluded. Urine cultures of these symptomatic patients were collected and antimicrobial therapy was given accordingly.

Along with the routine antenatal laboratory tests, patients were instructed to collect mid-stream urine samples. Collected samples were immediately inoculated on 5% sheep blood agar and eosin-methylene blue agar plates with a 0.01-mL loop. After 24–48 hours of aerobic inoculation at 37°C, the plates were interpreted. Colony counts of a single microorganism of >10^5^ colony forming units (cfu)/mL were interpreted as bacteriuria. The presence of multiple organisms or of skin flora was considered to result from contamination.

Antimicrobial susceptibility testing by disc diffusion was employed according to the Clinical and Laboratory Standards Institute Guidelines. Identification and antimicrobial susceptibility testing of microorganisms was performed according to the Clinical and Laboratory Standards Institute Guidelines with conventional microbiological methods and confirmed by The BD Phoenix Automated Microbiology System (BD Diagnostics, Sparks, MD). The Phoenix is designed for the rapid identification (ID) and antimicrobial susceptibility testing (AST) of clinically significant human bacterial pathogens. The Phoenix identification method uses modified conventional, fluorogenic, and chromogenic substrates. Research-use-only combination panels (UMIC/ID) for both the identification and susceptibility testing were used for this comparison. Software versions V3.34A and V3.54A were used for this study. The ID side contains 45 wells with dried biochemical substrates and 2 fluorescent control wells. The ID broth was inoculated with bacterial colonies from a pure culture that was adjusted to a 0.5 McFarland standard by using a CrystalSpec nephelometer (BD Diagnostics) according to the manufacturer's recommendations. A 25-*μ*L aliquot of this suspension was removed for AST, and the remaining suspension was then poured into the ID side of the Phoenix panel. The specimen was logged and loaded into the instrument within the specified timeline of 30 min. Quality control was performed according to the manufacturer's recommendations. The AST side of the combination panel contains up to 84 wells with dried antimicrobial panels and growth control well. The assay is a broth-based microdilution test. The system uses a redox indicator for the detection of organism growth in the presence of an antimicrobial agent. The previously described 25 *μ*L of the standardized ID broth suspension was transferred to the AST broth, yielding a final concentration of approximately 5 × 10^5^ CFU/mL. Quality control was performed according to the manufacturer's recommendations. 

The differences in the demographic features between the two groups were analyzed by the SPSS v14.0 Student's *t*-test.

## 3. Results

Demographic features of the women screened for ASB are displayed in Table 1. Age, gravid, and parity are similar between the two groups (*P* > .05). Hemoglobin levels, however, display a significant difference between the ASB and nonbacteriuria groups (*P* = .01).

Of the 2011 pregnant women included in the study, 171 (8.5%) had ASB. *E. coli*, detected in 131 (76.6%) patients, was the most frequently isolated organism, followed by Klebsiella pneumonia, which was found in 25 (14.6%) of the cases.  Table 2 shows the isolated microorganisms.

*E. coli*, the most common isolate, was found to be only 57.2% and 68% sensitive to ampicillin and amoxicillin + clavulanate, respectively. Its sensitivity fosfomycin was very high, 99.2%. Sensitivities to ceftriaxone and ciprofloxacin were both 90%, and sensitivity to amikacin was 99%. A 100% sensitivity was found for the broad spectrum penicillins imipenem and meropenem. An 86% sensitivity was documented for cefuroxime, a commonly prescribed oral agent for UTIs in pregnancy.

Klebsiella pneumonia, the second most frequent organism grown on the cultures, was only 4% sensitive to ampicillin, while its sensitivity to amoxicillin/clavulanic acid was 84%. We found a 88% sensitivity to fosfomycin. Sensitivity to cefuroxime was 84%, and a 100% sensitivity was found for cefepime, ceftriaxone, imipenem, and meropenem.

Antibiotic sensitivities of the isolated gram (−) and gram (+) microorganism are shown separately in Tables 3 and 4.

## 4. Discussion

Pregnant women with ASB are more likely to deliver premature or low birth weight infants and have a 20–30-fold increased risk of developing pyelonephritis compared to women without bacteriuria [9].

In this study, 171 of 2011 (8.5%) pregnant women had ASB, which is comparable with the results of studies by Uncu et al. [10] in 2002 and Hazhir [11] in 2007. *E. coli* was the most common isolate (76.6%), which is consistent with previous studies [12–14].

*E. coli*, the most frequent isolate, was 99.2% sensitive to fosfomycin. Fosfomycin is an oral agent administered in a single dose with relatively limited side effects. Klebsiella pneumonia, the second most common microorganism isolated in this study is, also 88% sensitive to fosfomycin.

Ampicillin and amoxicillin/clavulanate are two frequently prescribed oral antimicrobial agents for UTI in pregnant women. Our culture results yielded 57.2% and 68% sensitivities to these agents, respectively. Cefuroxime is another drug commonly prescribed for treating ASB, and sensitivity to this drug was 86%, which is comparable to that of cefazolin (87%), cefepime (91.6%), and ceftriaxone (90%). Sensitivities to imipenem and meropenem were both 100% (Table 3).

In some studies, gram-positive microorganisms are found to be important causative agents of ASB. Enayat et al. reported that up to 16.8% of causative organisms are coagulase-negative staphylococci [13]. In our study, only 5.8% of the urinary isolates were gram-positive organisms, the most frequent one being Enterococcus faecalis (4%).

 Contributing risk factors for developing bacteriuria during pregnancy are age, parity, sexual intercourse, diabetes mellitus, sickle cell disease, trait, anatomical abnormalities of the urinary tract, previous history of UTI, and socioeconomic status [15–18]. In this study, four variables, age, parity, and hemoglobin levels, were evaluated. Among these variables, hemoglobin levels were associated with ASB (*P* = .01). Because an association between bacteriuria and anemia has not been confirmed, we concluded that this result may be attributed to the confounding effects of low socioeconomic status.

Two of the most important points to consider in the choice of antimicrobial agents for use in a population of pregnant women within a certain geographical region are the frequently isolated urinary pathogens and the patterns of antimicrobial resistance.

## 5. Conclusion

Every symptomatic or asymptomatic woman should have a clean-catch urine culture, and detected cases should be treated according to the antimicrobial susceptibility tests. A clean-catch culture is the most sensitive test for the detection of ASB, but it is expensive and requires trained laboratory staff. When these facilities are not available, knowledge of the most frequent isolates and local antimicrobial resistance patterns will aid physicians in the successful empirical treatment of the infection.

In this certain geographical region, we found *E. coli* as the most common causative agent of ASB in the obstetric population and it is very sensitive to fosfomycin. We recommend fosfomycin for ASB in pregnant women due to its high sensitivity, ease of administration, and safety for use in pregnancy.

## Figures and Tables

**Table 1:** Demographic characteristics of the pregnant women screened for asymptomatic bacteriuria.

Characteristics	Non-Bacteriuria	Bacteriuria	*P* Value
Age (years)			
Mean (SD)	24.6 (5.1)	23.9 (5.0)	>.05
Range	17–43	17–44	
Pregnancy			
Mean (SD)	1.96 (1.0)	2.01 (1.1)	>.05
Range	0–6	0–7	
Parity			
Mean (SD)	0.86 (0.92)	0.91 (0.98)	>.05
Range	0–5	0–6	
Hemoglobin (g/dL)			
Mean (SD)	12.8 (6.8)	11.1 (5.3)	.01
Range	8.3–14.9	7.6–13.7	

SD = standard deviation.

**Table 2:** Bacteriologic isolates from pregnant women with asymptomatic bacteriuria.

Microorganism (s)	*N* (%)
*E. coli*	131 (76.6)
Klebsiella pneumoniae	25 (14.6)
Enterococcus faecalis	7 (4)
Klebsiella oxytoca	2 (1.2)
Proteus spp.	2 (1.2)
Enterobacter sakazakii	1 (0.6)
Enterococcus spp.	1 (0.6)
Staphylococcus aureus	1 (0.6)
Staphylococcus saprophyticus	1 (0.6)
Total	171 (100)

**Table 3:** Gram negative microorganisms.

	Amo/clav	Am	Cf	Cef	Ceft	Cefu	Fos	Fm	Sxt
Microorganism (s)	Antibiotics sensitivity (%)								
*E. coli*	89	75	115	120	118	113	130	125	108
	(68)	(57.2)	(87)	(91.6)	(90)	(86)	(99.2)	(95.4)	(82)
Klebsiella pneumoniae	21	1	23	25	25	21	22	9	25
	(84)	(4)	(92)	(100)	(100)	(84)	(88)	(36)	(100)
Klebsiella oxytoca	0	0	0	0	0	0	2	1	2
							(100)	(50)	(100)
Proteus spp.	2	1	2	2	2	2	2	2	0
	(100)	(50)	(100)	(100)	(100)	(100)	(100)	(100)	
Enterobacter sakazakii	0	0	0	1	1	1	1	1	1
				(100)	(100)	(100)	(100)	(100)	(100)

*Amo/clav=amoxicillin/clavulanic acid, *Am=ampicillin, Cf=Cefazoline, Cef=Cefepime, Ceft=Ceftriaxone,* Cefu=Cefuroxime, *Fos=Fosfomycin, Fm=Nitrofurantoin, Sxt=co trimoxazole.

**Table 4:** Gram positive microorganisms.

	Am	Fos	penG	Sxt	Clin	Eryt	Fm
Microorganism (s)	Antibiotics sensitivity (%)						
Enterococcus faecalis	7	7	6	ND	ND	ND	ND
	(100)	(100)	(85.7)				
Enterococcus spp.	1	ND	1	ND	ND	ND	ND
	(100)		(100)				
Staphylococcus aureus	ND	ND	0	ND	1	1	1
					(100)	(100)	(100)
Staphylococcus saprophyticus	0	ND	0	1	ND	ND	ND
				(100)			

Am=ampicillin, Fos=Fosfomycin, PenG=Penicillin G, Sxt=co-trimoxazole, Clin=Clindamycin, Eryt=Erythromycin, Fm=Nitrofurantoin, ND=not done.
